# The Horizons of Chronic Shame

**DOI:** 10.1007/s10746-022-09645-3

**Published:** 2022-10-14

**Authors:** Luna Dolezal

**Affiliations:** 1 Wellcome Centre for Cultures and Environments of Health, University of Exeter, Exeter, UK

**Keywords:** Shame, Chronic Shame, Phenomenology, Edmund Husserl, Horizons, Anticipation

## Abstract

Experiences of shame are not always discrete, but can be recurrent, persistent or enduring. To use the feminist phenomenologist Sandra Lee Bartky’s formulation, shame is not always an acute event, but can become a “pervasive affective attunement” (Bartky, 1990: 85). Instead of experiencing shame as a discrete event with a finite duration, it can be experienced as a persistent, and perhaps, permanent possibility in daily life. This sort of pervasive or persistent shame is commonly referred to as “chronic shame” (Pattison, 2000; Nathanson, 1992; Dolezal, 2015). Chronic shame is frequently associated with political oppression and marginalization. In chronic shame, it is the potentiality of shame, rather than the actuality, that is significant. In other words, the anticipation of shame (whether explicit or implicit) comes to be a defining feature of one’s lived experience. Living with chronic shame has important socio-political consequences. Thus far, chronic shame has eluded simple phenomenological analysis, largely because chronic shame often does not have a clear experiential profile: it is frequently characterised by the *absence* rather than the *presence* of shame. The aim of this article is to provide a phenomenology of chronic shame, drawing from Edmund Husserl’s formulation of the ‘horizon’ as a means a to discuss structural aspects of chronic shame experiences, in particular how chronic shame is characterised by structures of absence and anticipation.

## Introduction

While there are countless shame varieties and variations on shame experiences, most phenomenological accounts of shame rely on what might be considered a prototypical shame experience to make their analyses: a transgression of some nature occurs which is witnessed (literally or figuratively) by an Other; the consequence of this transgression is a shame reaction: a knowledge that one is somehow in the wrong; accompanied by a sense of intense exposure and a sense of wanting to withdraw or conceal oneself; this is further accompanied by a strong negative affective response, a feeling of emotional pain or anguish. This sort of prototypical shame moment is often characterised as “acute shame” (Dolezal, 2015; Zahavi, 2014). It is acute insofar as shame is experienced as a discrete reaction: an emotional event that arises, has a certain duration, and then dissipates.

However, experiences of shame are not always discrete, but can be recurrent, persistent or enduring. In her discussion of women’s experience of shame, the feminist phenomenologist Sandra Lee Bartky discusses how shame is not always an acute event, but can become a “pervasive affective attunement” (Bartky, 1990: 85). Instead of experiencing shame as a discrete event with a finite duration, it is experienced as a persistent, and perhaps, permanent possibility in daily life. This sort of pervasive or persistent shame is commonly referred to as “chronic shame” (Dolezal, 2015; Harris-Perry, 2011; Nathanson, 1992; Pattison, 2000: 93ff). Individuals who experience chronic shame have been variously described as “shame-bound” (Pattison, 2000: 93), “shame-prone” (Harris-Perry, 2011: 122), a “shame subjectivity,” (Berlant et al., 2008) or living “shame as a state of being” (Bradshaw, 1988: vii).

Chronic shame in Bartky’s account and elsewhere (e.g., Harris-Perry, 2011) has a strong political valence; it is a symptom of systematic oppression and social domination. However, not all experiences of chronic shame arise from socio-political circumstances, and research suggests that susceptibility to chronic shame in adult experience can be caused by a variety of factors, including childhood abuse or dysfunctional shame-based family dynamics (Pattison, 2000), relational trauma in infancy (DeYoung, 2015), as well as being a feature of certain psychopathologies such as body dysmorphic disorder (Phillips, 2005), PTSD (Herman, 2011; Taylor, 2015) or anxiety disorders (Fergus et al., 2010).

Chronic shame is usually described in contradistinction to acute shame, where the two varieties of shame experience have differing phenomenological profiles. In experiences of chronic shame, shame is not attached to a particular episode or instance of transgression; instead, shame becomes global and diffuse, causing a continuous sense of social anxiety, personal inadequacy and relational disconnection. In chronic shame, shame becomes experienced less as a discrete emotional episode, but instead, following Bartky, as “a profound mode of disclosure both of self and situation” (Bartky, 1990: 85). Chronic shame in Bartky’s account is profoundly political; it is a symptom of systematic patriarchal oppression and social domination. While not all experiences of chronic shame arise because of political structures or power imbalances, I will argue that living with chronic shame is inherently politically compromising. Hence, understanding chronic shame and its phenomenology is of particular importance.

However, chronic shame has eluded simple phenomenological analysis. In part, the difficulty arises because chronic shame is not one clear concept, nor one unified and consistent experience. Following Pattison, I take the term ‘chronic shame’ to be most usefully understood as a socially-constructed explanatory concept which captures a dimension of lived experience that has a range of intensities and manifestations (Pattison, 2000: 95). While there are other terms that could be used to describe the phenomenon of shame as a ‘pervasive affective attunement,’ (e.g., ‘shame-proneness,’‘dispositional shame,’ or being ‘shame-bound’), in this article I will use the term ‘chronic shame,’ as this is the term that I have found most consistently across various literatures.

The underlying commonality in experiences understood as ‘chronic shame’ is a persistent and enduring ‘attunement’ to the experience of shame, which is described in the various literatures as manifesting in experience in two dominant manners: explicit and implicit. While I offer this conceptual demarcation between explicit and implicit chronic shame in this paper, I want to acknowledge that these two experiences of chronic shame are by no means completely distinct, and the conceptual demarcation I offer must be considered simply as such. I separate these types of chronic shame largely for the sake of conceptual clarity and discussion. The reality is that in lived experience, emotions are overlapping, complex and often not easy to parse. The complexities and realities of an individual’s experience of chronic shame, across time and within varying circumstances, may overflow the categories which I will offer here. Despite this, I believe these conceptual demarcations will be useful.

First, explicit chronic shame is described as an experience of having a conscious and persistent expectation that acute shame is an imminent possibility. As a result of this *explicit* anticipation of shame, one structures one’s behaviour and interactions around avoiding shameful exposure.^1^ Second, chronic shame is also described as being *implicit* in one’s experience. As Bartky notes in her discussion of chronic shame in women’s experience, one’s attunement to shame is often “less available to consciousness and more likely to be denied” (Bartky, 1990: 85). This means one may not even be aware that one is chronically anticipating shame or shameful exposure; or it may mean that one may not be willing to identify one’s experience as dominated by shame (perhaps because this would be too painful, shameful or psychologically threatening). Instead, one’s experience may be dominated by strategies to ‘bypass’ shame, or ‘compensate’ for shame. For many, implicit chronic shame involves living not with shame, but with a pervasive sense of inadequacy, insecurity and inferiority. Nonetheless, in implicit experiences of chronic shame, one’s experience is still structured around avoiding shameful exposure, even if this remains largely outside of conscious awareness. In both the explicit and implicit cases, the anticipation of shame (whether this anticipation is explicit or implicit) comes to be a defining feature of one’s lived experience.

Hence, living with chronic shame does not mean that shame is continuously experienced, but instead that the threat of shame is more predominant and persistent. In *explicit* chronic shame, this ‘threat’ is registered on a cognitive and emotional level; one is consciously and affectively attuned to the possibility of acute shame arising in a moment of shameful exposure. In cases of *implicit* chronic shame, this ‘threat’ may not register in conscious awareness, but nonetheless serves to organize one’s behaviour, disposition and interactions.

Both explicit and implicit chronic shame are frequently characterised by the *absence* rather than the *presence* of shame. In these experiences, in differing manners, shame becomes a negative space around which experience is organized. Instead of the very recognizable searingly painful self-consciousness that accompanies episodes of acute shame, chronic shame can render shame invisible through implicit and explicit shame avoidance strategies. In this way, shame can become invisible within social interactions and sometimes even to the self who is ‘experiencing’ it. As a result, as I have suggested elsewhere (Dolezal, 2020), there are inherent challenges to identifying, diagnosing and describing the experience of chronic shame, and, concomitantly, attempting to describe its phenomenology. My aim in this paper is to begin a phenomenology of chronic shame, addressing both explicit and implicit varieties of this experience. I will do so through drawing from some conceptual tools within Edmund Husserl’s phenomenology, namely engaging with his formulation of the ‘horizon’ as a means a to discuss structural aspects of chronic shame experiences. In particular, I will consider how chronic shame is characterised by structures of absence, anticipation and intersubjectivity, while playing a role in the formation of the character of one’s lifeworld.

## Shame

First, let us begin by understanding shame. Shame is commonly characterized as a negative self-conscious emotion; it is an experience that arises when we are concerned about how we are seen and judged by others. It involves self-consciousness because for every shame experience the object of shame is oneself and, furthermore, this self-consciousness is mediated through an awareness of how other people may view the self. Structurally, shame involves three components: the self, the other, and a norm or standard that has not been met. In general terms, shame arises when the self is witnessed by the other transgressing, or failing to live up to, the norm, rule or standard in question. We feel ashamed when we are seen by another or others (whether they are empirically present, merely imagined or a viewpoint that has been internalized) to be flawed in some crucial way, or when some part of our core self is perceived to be inadequate, inappropriate, or immoral. As Sartre puts it: “Thus shame is the unitary apprehension of three dimensions: *‘I am* ashamed of *myself* before *the Other*’” (Sartre, 2018: 393). Commonly distinguished from guilt, where we feel bad about an action or something that we have *done,* shame is about the person that one *is*. Shame is a powerful experience that can feel immensely threatening to the self. During a shame experience, we feel deeply and often irreparably flawed. When shame arises, we can feel that we are unworthy and unlovable, and that our social position and our social bonds are under threat.

While shame takes many forms, acute shame—a discrete emotional reaction to an event where a transgression by the self has been revealed to an ‘Other’—serves both philosophically and commonsensically as the prototypical shame experience (Zahavi, 2014: 239). Jean-Paul Sartre’s discussion of shame in *Being and Nothing-ness* furnishes us with examples of acute shame, illustrating the tripartite structure of shame. Sartre writes, “I have just made some clumsy or vulgar gesture: this gesture sticks to me; I neither judge it or blame it; I simply live it … But now, all of a sudden I raise my head: somebody was there, and has seen me. All at once I realize the vulgarity of my gesture in its entirety, and I am ashamed” (Sartre, 2018: 308). In Sartre’s well-known voyeur vignette, the voyeur recounts: “there I am, bent over the keyhole; suddenly I hear some steps. A shudder of shame runs through me: someone has seen me. I stand, and I scan with my eyes the deserted corridor: it was a false alarm” (Sartre, 2018: 377). When we think of shame, we usually think of discrete events such as these where shame occurs suddenly as a reaction to someone witnessing a transgression, mistake or misdeed on our part, even if that ‘someone’ is imagined, mistaken or absent.

Acute shame has a typical phenomenological profile. In general, one is over-whelmed physically, and common physical responses include a sense of intense physical exposure, coupled with a sense of wanting to hide or withdraw (Kaufman, 1993). In moments of shame the body folds in on itself. Often one’s posture is stooped, the gaze is downward, the head is bowed; we want to shrink or get smaller, or to just disappear. There are many other possible physical signs of shame, such as blushing, stuttering, sweating, blanching, hesitating, cowering, covering the face, a sinking feeling (Goffman, 1967: 97). With these physical and physiological responses is an intense feeling of emotional anguish or pain. There is a sinking feeling of dread, coupled with an acute anxiety and self-consciousness focused on some negative evaluation of the self and a concern with how the self is or will be perceived by others. Ultimately, the fear behind shame is rooted in concerns about belonging and acceptance. Shame signals to us that our social bonds are under threat and that we may be rejected or ostracized (Scheff, 2000). As a result, shame is alienating, isolating and deeply disturbing. It can provoke powerful feelings of despair, inferiority, powerlessness, defectiveness and self-contempt, to name a few.

The extreme negative self-consciousness that comes with shame, coupled with its intense physical response, gives shame its most recognizable feature: the feeling that one is completely, and uncontrollably, exposed. In shame, there is the certainty that everyone can see your flaws and mistakes, and from that comes a further desire to hide or disappear. This exposure leads to a sense of paralysis as a result of feeling intensely conspicuous, or what Kaufman terms “binding”. Kaufman writes: “Exposure can interrupt movement, bind speech and make eye contact intolerable. Shame paralyzes the self” (Kaufman, 1993: 5, 18). Zahavi likewise discusses how “the acute experience of shame might give rise to something akin to bodily paralysis” (Zahavi, 2014: 222). The metaphor of being caught exposed at the end of a zoom lens in a camera is commonly deployed to describe this experience (Brown, 2010: 68). One freezes because of the sense that one is isolated, singled-out and profoundly conspicuous. There is a strong feeling of vulnerability that comes with this sense of paralysing exposure. It feels as though everyone can see your flaws or misdeeds and that they may scorn, judge and reject you.

## Chronic Shame

In contrast to experiences of acute shame, chronic shame has a very different phenomenological profile. As Pattison notes:

There is an enormous difference between acute, reactive shame and the chronic shame that shapes a whole personality and may last a lifetime. When individuals appear to experience the whole of life as actually or potentially shame-productive and manifest such symptoms as withdrawal, self-contempt, inferiority and gaze aversion as a matter of course throughout their everyday lives, shame has become pathological and chronic. (Pattison, 2000: 83)

While chronic shame shares many of the painful features of acute shame, such as emotional pain, self-consciousness, a sense of visibility, it is not experienced as an acute reaction of emotional torment and hyper-self-consciousness. Acute shame often happens unexpectedly, and takes the subject by surprise. In contrast, chronic shame is charaterised by a persistent presence of shame’s possibility. However, it must be stressed that chronic shame is not a state of perpetually feeling ashamed (as we might experience chronic pain as a constant pain state).

In what follows, I offer two examples as case studies to illustrate the phenomenological profile of chronic shame. The first example focuses on the experience of *explicit* chronic shame that is intimately connected to a politicized identity and the consequences of stigma. In this example, the chronic anticipation of shame leads to a persistent and heightened conscious awareness of the possibility of shameful exposure in daily life. The second example focuses on the experience of *implicit* chronic shame, where the anticipation of shame is not fully present to conscious life. As Bartky describes, the attunement to shame in these cases is “less available to consciousness and more likely to be denied” (Bartky, 1990: 85). I offer these examples in order to illustrate the structure of the ‘present absence’ which, I argue, can be useful to elucidate phenomenologically both explicit and implicit varieties of chronic shame.

To illustrate explicit chronic shame, I offer an example here from queer theorist and scholar Kane Race.^2^ He writes eloquently in a semi-autobiographical essay about his experience of receiving an HIV diagnosis in 1996, the same year that combination anti-retroviral therapy transformed HIV from a death sentence to a chronic and manageable illness:

Soon after receiving my HIV diagnosis I made an appointment with a general practitioner with years of specialist experience in HIV care … “Don’t tell your parents,” was the first piece of advice he offered me … I did hold off on telling my parents for some time … But living in constant fear of imminent exposure with a stigmatized, sexually humiliating condition takes its toll … It was only a matter of time before they discovered my dirty secret, I figured. In the narrative that dominated my parental imaginary, HIV operated as vindicating proof of sexual depravity: conclusive evidence of the wrongness of homosexuality and the foolish lifestyle I was leading. I imagined my parent’s anger, disappointment, and deep shame on discovering that their only son had succumbed to the logical outcome of the homosexual lifestyle they had warned me against repeatedly. I had wasted my life, wasted everything, betrayed every hope they had invested in me. This apprehension of a wasted life filled me with a sense of impending catastrophe … Intensely conscious of the shameful disappointment I had become, I lived in constant fear of public humiliation and unwanted exposure … [as a result of the] intense sexual shame that the possibility of having my HIV status outed to my parents unwittingly provoked in me. (Race, 2021)

Race’s testimony discusses explicitly how the shame associated with this diagnosis and its inherent relation to his sexuality came to dominate his experience. A ‘constant fear’ of exposure and of ‘impending catastrophe’ was intimately linked to a negative evaluation of the self through the eyes of the Other, in this case the ‘Other’ was dominated by his ‘parental imaginary’. Race describes how shame is anticipated: at any moment, humiliating exposure is possible: he may be judged for his ‘sexual depravity’ and subsequently rejected. The explicit anticipation of shame, related both to his sexuality and HIV-status, comes to be a defining feature of his lived experience (Dolezal, 2021, 2022).

To illustrate *implicit* chronic shame, I draw from Bartky’s analysis of women’s shame in her essay “‘Shame and Gender’” (Bartky, 1990). Her interest in this essay is to look at the “most pervasive patterns of gendered emotion” in order to consider how emotion becomes part of the “phenomenology of oppression,” where “women are not just situated differently than men within the social ensemble, but are actively subordinated to them within it” (Bartky, 1990: 84). Bartky discusses a case study of a classroom of high school teachers, and her experience, as the teacher, of some of the gendered differences in terms of how women (versus men) spoke, handed in assignments and engaged in the classroom in general. The womens’s behaviour, as she describes it, was characterised in general by a lack of self-confidence, insecurity and feelings of lacking authority or credibility. As she notes, they behaved as though they were “ashamed” of their written work and of expressing their ideas and that an attunement to shame coloured their comportment and speech (Bartky, 1990: 89):

It seems to me that the demeanor of my female students in that suburban class-room bore the characteristic marks of shame, of a shame felt directly or anticipated: In their silence, the necessity for hiding and concealment; in the tentative character of their speech and in their regular apologetics, the sense of self as defective or diminished. The fear of demeaning treatment could be seen in the cringing before an Other from whom such treatment was anticipated; shame could be read even in the physical constriction of their bodies. (Bartky, 1990: 90)

Bartky uses the term “shame” as a means to make sense of this behaviour of subordination, where shame is not to be understood as an acute emotion experience but rather as an “emotional attunement” that unites all of these traits and beliefs about the self (Bartky, 1990: 83). She writes: “The shame of some of these women was not a discrete occurrence, but a perpetual attunement, the pervasive affective taste of a life” (Bartky, 1990: 96). The experience of shame as an emotional attunement that Bartky describes is not explicit. The women in her classroom, she argues, would not have identified themselves as experiencing or anticipating shame, nor would they have agreed with the suggestion that they felt shame about their classroom work: “My students felt inadequate without really believing themselves to be inadequate in the salient respects: They sensed something inferior about themselves without believing themselves to be generally inferior at all” (Bartky, 1990: 93). Shame, for these subjects of subordination, to reiterate Bartky’s claim, is “less available to consciousness and more likely to be denied” (Bartky, 1990: 85). Nonetheless, as Bartky’s account makes clear, even when the anticipation of shame or shameful exposure is implicit (that is, outside of a subject’s conscious experience), shame can still be identified as an organizing principle of one’s life and lifeworld. To reiterate a point made above, avoiding shame becomes a central feature of subjectivity, and an absent, but anticipated, shame becomes the negative space around which experience is organized.

## Chronic Shame as a Present Absence

As both Race’s and Bartky’s accounts demonstrate, living with chronic shame does not mean that shame is continuously experienced, but instead that shame avoidance becomes a dominant feature of experience. Race’s account shows how in experiences of explicit chronic shame, acute shame is incessantly anticipated: at any moment, he expects a moment of humiliating exposure or a moment of dehumanizing objectification. Writing about explicit chronic shame, Pattison notes, “Those whose lives are shaped by persistent shame attitudes and reactions … feel, or act as if they feel, that shame may afflict them at any moment” (Pattison, 2000: 83). However, because acute shame is so painful and potentially threatening to one’s sense of self and one’s social bonds, it is often mostly avoided or circumvented. Pattison continues: “They live their lives trying to avoid occasions and relationships that might provoke painful shame experiences” (Pattison, 2000: 83). Individuals experiencing explicit chronic shame come to behave in an “anticipatory way” with respect to shame, “adjusting present behaviour in order to address future problems” (Poli, 2010: 8). In experiences of explicit chronic shame, acute shame can be fastidiously avoided through controlling one’s behaviour, interactions and actions. The persistent avoidance of acute shame is characteristic of explicit chronic shame experiences.

In contrast, in experiences of implicit chronic shame, such as those described by Bartky, shame avoidance is not realised through conscious strategies, but often unconsciously through socialised habits of compartment. While individuals are also continually avoiding shame and shameful exposure, this may be something they don’t even realise that they are doing routinely or with regularity. To return to Bartky’s analysis, the women in her classroom avoided shameful exposure—the implicit anticipation of being shamed by ‘Others’ or their teacher for what they perceived to be inferior work or ideas—through silence, through apologies, through hesitation. In addition to these strategies of shame avoidance, in experiences of implicit chronic shame there is another layer of shame avoidance: individuals do not just avoid shame in experience, but avoid shame altogether; it becomes bypassed for other experiences. This is what can render it *implicit*.

For instance, In her book *Understanding and Treating Chronic Shame*, DeYoung discusses how in psychotherapeutic treatment shame often “disappears” (DeYoung, 2015: 24). This happens “precisely because [shame] is so very painful to bring to awareness … instead of feeling the emotional intensity of shame itself, a client may turn either to obsessive self-hatred or obsessive thoughts about what went wrong in interactions between self and others” (DeYoung, 2015: 24). DeYoung describes chronic shame as “silent,” shadowing her clients’ experiences in the world:

Clients who struggle with the disintegrating power of chronic shame may not daily or consciously expect to be annihilated by shame. However, the threat is always around somewhere, just out of awareness, kept at bay. What they live with daily is what it costs them to keep from falling into shame. (DeYoung, 2015: 19)

DeYoung argues that some of her clients who suffer from chronic shame do not even know that they are anticipating shame (and related strategies to circumvent the threat of shame) with debilitating frequency. The anticipation of shame in these cases works through “implicit anticipations” which “work below the level of consciousness” (Poli, 2010: 12). Not only is shame absent in experience, but its anticipation is also characterized by an absence. In these cases shame is avoided through psychological coping mechanisms such as denial or “defensive scripts” (Pattison, 2000: 111) where shame is “bypassed” (Lewis, 1987) for other experiences, such as depression, apathy, narcissism, rage, perfectionism, withdrawal or anger, among others (Nathanson, 1992; Pattison, 2000).^3^ As Bartky notes in her discussion of politicized implicit chronic shame, oppressed minorities may never identify their experiences as chronic shame and instead may experience this attunement to shame manifested as personal inadequacy, rather than as the express result of unjust domination or subordination (Bartky, 1990: 85).

As a result, chronic shame is an experience of shame, which nonetheless often involves avoiding shame or bypassing shame. Through parsing out the difference between explicit and implicit chronic shame, I have identified two ways which shame avoidance happens. First, shame is anticipated, but remains absent through strategies of shame avoidance. These strategies can be both conscious and unconscious, and they are used to manage behaviour, comportment, actions and interactions to ensure that shame does not arise. Second, shame avoidance happens through the bypassing of shame. In these cases, one does not even realise that shame avoidance is part of one’s experience, and shame goes thoroughly ‘underground,’ with other experiences, such as self-hatred, anger, excessive pride, insecurity, for instance, being experienced in its place. Understood in this way, in general terms chronic shame can be characterized as a persistent *absence of shame* (whether it is anticipated shame or bypassed shame), which is nonetheless experienced *as present*. What this means is that the absence of shame structures present experience. And this ‘present absence’ has tangible consequences in terms of how one experiences and organises one’s behaviour, action, social relations and broader world.^4^

Understanding chronic shame through the conceptual tool of a ‘present absence’ gives us inroads to describing the experience phenomenologically. In what follows, I will turn to theoretical resources in the work of Edmund Husserl in order to undertake a phenomenological analysis of the lived experience of chronic shame, unpacking aspects of the examples above through the theoretical resources in Husserl’s work. In particular, Husserl’s concept of the ‘horizon’ will be examined and utilized as a means to articulate some of the structural features of chronic shame related to absence, anticipation, intersubjectivity and the character of one’s lifeworld.

## The Horizonal Structure of Experience in Husserl’s Phenomenology

Husserl’s seminal descriptions of the experience of consciousness demonstrate that all human experience is characterized by experiences of ‘present absences’: as he puts it, what is “strictly non-experienced but necessarily also meant” (Husserl, 1977: 23). Husserl uses the concept of the ‘horizon’ to capture this idea, borrowing meaning from how we understand horizon in the usual sense as the limit of what is visible or perceptible (Geniusas, 2012: 1). When we look out over the sea to the edge of what is visible, the horizon isn’t a fixed location, but a shifting limit of possible experience. We will never arrive at the horizon, it is always repositioning itself relative to our own location. Nonetheless, the horizon acts experientially as a real limit point demarcating what is experienced (that which is currently visible) from what is ‘non-experienced’ or a ‘non-presence’ but nonetheless within the realm of possibility and hence in some sense part of experience: we know that beyond the visible horizon there is more available to experience; there is always more ocean to see, or land to explore.

Hence, “horizons,” for Husserl, “are ‘predelineated’ potentialities” in our experience (Husserl, 1977: 45). He notes that in lived experience, “*every actuality involves its potentialities*, which are not empty possibilities, but rather possibilities intentionally predelineated in respect of content … having the character of possibilities *actu-alisable by the Ego*” (Husserl, 1977: 44).^5^ The case of external perception of spatial objects serves as paradigmatic for Husserl in explicating the idea of horizons in the experiences of consciousness more generally (Husserl, 2001: 57). The perception of a spatial object in external perception involves a constant relationship between presence and absence, or “fullness and emptiness” to use one of Husserl’s formulations (Husserl, 2001: 44). Husserl explains, external perception of an object “is only possible in the form of an actually and genuinely original conscious-having of sides and a co-consciousness of other sides that are precisely not originally there … the non-visible sides are certainly also there somehow for consciousness, ‘co-meant’ as co-present” (Husserl, 2001: 40).

In short, what Husserl describes is that in external perception objects present themselves in an adumbrated, or many-sided, manner where the sides of an object which are visible in a given moment simultaneously indicate the other aspects of the object which are not presently visible. These absent sides are ‘co-present’ insofar as they are included in the horizon of possible perceptual experience. Husserl writes:

[E]verything that genuinely appears is an appearing thing only by virtue of being intertwined and permeated with an intentional empty horizon, that is, by virtue of being surrounded by a halo of emptiness with respect to appearance. It is an emptiness which is not a nothingness, but an emptiness to be filled-out; it is a determinable indeterminacy. (Husserl, 2001: 42)

For instance, when I look at a tree, there is no way for me to see it all at once. Instead, by necessity, I can only see one side of its trunk and foliage in my perceptual field; the other side is absent to my current perception. However, as part of the horizon of possibilities that my perception of the tree entails, the absent side of the trunk is also ‘present’ in my perception, but present as an ‘emptiness’ that needs to be ‘filled-out’. As Dan Zahavi notes, “it is only because of its [an object’s] embeddness in a horizon (of absence) that the present profile is constituted as a present profile” (Zahavi, 2003: 97). Often, in the case of external perception, this filling out is ‘actualizable by the Ego,’ to use Husserl’s formulation, simply by moving around the object: walking to the other side of the tree.

It is important to note that a horizon, understood as a co-present emptiness in an experience, is not something that is ‘imagined’ or ‘visualized’ by consciousness; horizons, Husserl makes clear, are distinct from “anticipative ‘imaginings’” (Husserl, 1977: 45). We don’t need to imagine or visualize the other side of the tree as part of the perceptual process. Instead, our perception is inherently characterized by this constant blending of what is presently available to perception, along with what is absent but potentially realizable. Perception, as one instance of conscious experience, is a constant play of absence and presence: as Husserl notes, “perception and non-perception continually pass over into one another” (Husserl, 1964: 62). The “accomplishment of consciousness” (Husserl, 2001: 57) is to synthesize the flow of presences and absences—as horizons expand and recede—into experience that is coherent and sensible. Perceiving a tree, hence, involves the perception of a unified tree-object through time, rather than the experience of a bunch of discrete, partial and disjointed tree impressions that need to be pieced together through some sort of cognitive process (Drummond, 2003).

While Husserl draws on the example of external perception of spatial objects as a way to illustrate how horizons operate in lived experience, horizons are part of our intentional experience of all manner of “objects,” “no matter what category of object they may be” (Husserl, 2001: 57). An object, for Husserl, is a very broad idea, essentially understood as anything at all about which something can be said (Moran & Cohen, 2012: 228). In other words, horizons help us make sense of what is unfolding in *all aspects* our experience through time,^6^ filling in what is ‘absent’ with what is already known or familiar, through a meaningful anticipation.

## The Horizonal Structure of Chronic Shame

When thinking through how chronic shame is experienced, as a persistent anticipation (whether implicit or explicit), yet absence, of acute shame, Husserl’s formulation of horizons as what is “strictly non-experienced but necessarily also meant” (Husserl, 1977: 23) becomes very useful. We can talk about the presence of shame in experiences of chronic shame, but only insofar as shame is understood to be a ‘present absence’. In the case of explicit chronic shame, shame avoidance strategies mean that acute shame remains on the horizon, it is ‘present,’ but remains ‘absent’ insofar as it is never fully realised, because one is so adept at avoiding circumstances or interactions where it might occur. In the case of implicit chronic shame, the ‘absence’ is multi-layered, as shame is not only avoided in interactions, but the very possibility of shame remains unacknowledged. However, it should be noted that in implicit chronic shame, where shame is bypassed and goes ‘underground,’ there is always the possibility that one will acknowledge shame at some future time. Through therapy, personal insights or research, an individual may come understand their experience to be characterised by chronic shame while this may not have been useful or possible for them before.

To return to Husserl’s language of horizon, in chronic shame, shame is ‘non-experienced,’ but ‘necessarily also meant’. This ‘present absence’ structures experience; one’s expectations, feelings, thoughts, actions and social relations are impacted by the shame which is avoided, or which exists ‘just out of awareness’. While some individuals may not even realize that shame is an enduring horizon of their experience (it is implicit), as Bartky and DeYoung describe, other individuals, like Race, may live with an acute awareness, or anxiety, of the possibility of shameful exposure (it is explicit).

In fact, Race’s testimony gives insight into how chronic shame can be experienced less as a ‘silent’ undercurrent, but as a persistent “shame anxiety” (Dolezal, 2021): an “anticipatory anxiety about the imminent threat of being exposed, humiliated, belittled or rejected” (Pattison, 2000: 85). In experiences of explicit chronic shame, the immediate future of one’s lived experience comes to be chronically overshadowed by the possibility of shame: acute shame is expected imminently. The horizons of one’s present experience come to be dominated by the possibility of shame; it feels as though shame is constantly just around the corner, or just over the horizon, so to speak. While the elucidation of horizons in the example of external perception of spatial objects, discussed above, emphasises the unfolding of spatial horizons, the structure of anticipation that occurs in chronic shame is best illustrated through an emphasises on its temporality. Shame is not lurking around a physical corner, but rather, lurking, temporally, in a near future reality that is anticipated (whether implicitly or explicitly) in the present. Hence, Husserl’s account of the temporal horizons in his writing on the phenomenology of internal time-consciousness are elucidatory in terms of understanding the structure of this anticipatory experience.

As Husserl notes, it is the temporal character of lived experience, or what he calls ‘internal time consciousness,’ that is the basis by whichcontains a “temporal halo” of what is “just past,” the unities experienced by consciousness become possible. This is due to the fact that we experience objects, such as the perception of the tree discussed above, with temporal breadth and we do so without the need to use memory or any sort of representation or imagination. As Husserl elucidates, consciousness renders sensible, or coherent, our experience and this occurs necessarily *through time*. In the case of perception of an external object, all spatial horizons have a temporal character; they extend both into the past and into the future. Every moment of perception in the “now” contains a “temporal halo” of what is “just past,” or “retentions,” along with the “expectation,” or “protentions,” of what is about to come (Husserl,1964: 58). Retentions and protentions constitute temporal horizons in our lived experience where every moment of experience is part of a continuum of the receding past and the anticipated future. In the case of external perception of a spatial object, the tree is a coherent unity, because I can hold on to my retentions of what I have *just seen*, and continue to anticipate what is *expected to be seen* in the coming moments. It is through internal time consciousness that the function of weaving together the current sensuous impressions with the horizons of what has come before and what is yet to come, becomes possible.

Hence, anticipation of what is presently absent in experience—through the protentional horizonal character of time consciousness—is, in fact, a structural part of any lived experience. In all experience we anticipate what is presently absent, but imminently expected, and this happens without the need for explicit cognition or any sort of conscious projection, imagination or expectation. It should be noted that this structure of horizonal anticipation occurs across a variety of temporal scales, from the “microanticipations” that occur in perceptual processes to longer forms of “social anticipation,” ranging from seconds, to minutes to days or years (Poli, 2010: 13). New impressions are constant fulfilments or disappointments of our anticipations, or protentions.

In general, the horizonal structure that Husserl elucidates serves as a way to understand how we make sense of our constantly varying experiences, or, in other words, “the capacity of consciousness to enwrap appearance within the structure of familiarity” (Geniusas, 2012: 8). Although horizons indicate an ‘emptiness’ to be ‘filled,’this ‘emptiness’ is not without content. For instance, once I am familiar with the tree-object, having perceived thousands in my lifetime, the horizons of possibilities regarding the tree’s appearance become narrower: I am sure ‘I know,’ without having to actually see, what the other side of the tree looks like. In this way, consciousness fills in the gaps in our experience and populates our protentions with anticipations of particular sorts.

Through repetition, familiarity and habit, or “sedimentations of experience” (Geniusas, 2012: 6), some anticipations or possibilities for fulfilment become more “enticing” than others; as Husserl discusses, they “appear with a different weight” (Husserl, 2001: 84). With familiarity, the “leeways of possibilities” (Husserl, 2001: 87) are reduced in scope. The ‘emptiness’ indicated by the horizon is in fact more readily filled in with what is familiar and, hence, ‘expected’. As a result, the sedimentations of experience “enable consciousness to project meaning into what is directly perceived” (Geniusas, 2012: 6). In short, my anticipations are tugged into the future in a particular direction that is based on what is familiar, known, habitual or sedimented in my experience.

Hence, we can start to understand how in the experience of chronic shame, the expectation or anticipation of shame can become a sedimented part of experience without the explicit need for any imagining, or conscious projection into the future. If all or many previous experiences have been infused with the possibility of shame, it follows that an atmosphere of shame, or an ‘affective attunement’ to shame, comes to dominate the horizon of what is possible in experience as it unfolds through time. As DeYoung argues, chronic shame develops when “many repetitions of … shame experience form a person’s lifelong patterns of self-awareness and response to others” (DeYoung, 2015: 18). While DeYoung’s account primarily articulates experiences of chronic shame which arise out of relationships of disintegration and dysregulation in early infancy and childhood (hence her emphasis on ‘lifelong’ patterns), her reasoning holds for experiences of chronic shame that develop in adult experience as a result of a having a marginalized identity: contracting HIV as a gay man in the 1990s, as per Race’s example, or being a woman subordinated within patriarchy, as per Bartky’s example. Repeated experiences of being shamed, or witnessing others like yourself being shamed, or sensing patterns of shaming across the population to which you belong, leads to a pattern of awareness, or a pattern of unconscious biases (Bartky, 1990: 93), where there is a chronic anticipation of shame in relation to one’s own experience and in relation to encounters with others.

Encounters with others are always part of the horizon of our possible experience, as the lived subject in Husserl’s account is always and already in a shared world horizon. As Husserl notes, we are all part of the “world of men” [sic], constituted with others, where for “each man [sic], every other is implicit in this [open undetermined] horizon” which constitutes “my actual and possible experience of the world” (Husserl, 1977: 130f). However, in experiences of chronic shame, the character of some, or many, others in my shared world horizon may come to be dominated by a suspicious, judgemental and objectifying other. As Bartky points out the “other” in experiences of chronic shame often remains unspecific and more like a “composite portrait” of the people, social forces and biases which have historically subjected one to “demeaning treatment” (Bartky, 1990: 90). Because of repeated patterns in social experience, one anticipates that the others that come into their world horizon, or at least some particular others (for example, one’s parents or one’s classroom teachers) are perceived to be primed to judge, scorn or shame.

While it may be the case that a judgemental other and an incident of shameful exposure is, in fact, just around the corner; it is also possible (and possibly more often the case) that the horizon of shame may never be fulfilled. It may be that one is mistaken about anticipating shame: there is in fact no judgmental or objectifying other lurking. Hence, while shame may dominate the horizon of experience, and we may feel ‘certain’ it is just around the corner, as Husserl makes clear, we can be mistaken by our anticipations; we may be expecting one thing but in fact quite a different thing comes to pass. Husserl offers an example of how we can construct subjective certainty from mere potentiality:

[A] cloudy sky together with humidity speak in favour of a thunderstorm, but not ‘for sure’. It entices in this way, and it does so in varying degrees, changing according to the particular circumstances … [I can] straightforwardly yield to the inclination … I definitively ‘decide’ for this possibility. I believe, I am ‘subjectively certain’ that there will be a thunderstorm, and fetch my raincoat and my umbrella. (Husserl, 2001: 85f)

With enticing possibilities, we are tugged into the future with a particular expectation. This expectation may be fulfilled (a thunderstorm breaks), or it may be mistaken (the clouds clear and the sun comes out), but nonetheless the character of the empty horizon in the present takes on a particular character or sense. In explicit chronic shame, one may come to feel ‘subjectively certain’ that shameful exposure is imminent, that others are looking, judging and objectifying; one is about to be rejected or scorned. This is precisely the experience described by Race in his essay. The experience of “living in constant fear of humiliating exposure—of trying to remain socially as well as virologically undetectable” (Race, 2021) because of the stigmas associated with HIV and homosexuality, led him to expect, and perhaps to feel ‘subjectively certain,’ that his parents would feel ‘anger, disappointment and deep shame’ upon learning of his HIV status. In fact, quite a different experience came to pass. Race writes:

It took me a decade to muster the courage to finally come out to my parents about my HIV status … As it happens, my parents’ response was unexpectedly generous and immensely reassuring. “You poor bastard,” my father said without the slightest hint of moral judgment. “What a terrible thing to live with, what a terrible accident”. By 2006 the long-term effectiveness of antiretroviral therapy had been well enough established to make a new script for HIV disclosure normatively available. (Race, 2021)

Indeed, through a shifting context of signification, the horizons of Race’s chronic shame shifted. Race was gradually able to dismantle the “forms of enforced silence, stigma, and fears of exposure” that dominated his existence, while recognizing that for many this may not be possible for a great many people living with HIV (Race, 2021).

What this example points to is the malleability and changeability of our experiential horizons. Chronic shame experiences are by no means static or stable, but can grow or lessen in intensity depending on context and circumstances. The anticipation of shame in one’s experiential horizon may be paralyzingly present in one context, and diminish to almost nothing in another. Race himself acknowledges his shifting thresholds for shame as he moves in and out of certain communities or contexts: “With an HIV-positive boyfriend and friends and colleagues in the sector, I was fortunate enough to have access to a support network that made it possible to avoid a painful family drama during the early stages of learning how to live with this disease” (Race, 2021). The shifting normative frameworks of our various “worlds” can shift the contours of our shame experiences (Lugones, 2003; Ortega, 2016).^7^ In other words, the degree to which we are anticipating shame, or willing to acknowledge shame, may depend on the ‘world’ we are inhabiting, and the normative standards of that world by which we feel we are being evaluated or judged.

## Socio-Political Considerations

The example of chronic shame via Kane Race’s theorizing of his experience of living with HIV has been useful for illustrating the phenomenological structures of chronic shame, via horizons and anticipations. However, it must be reiterated that this example is a case of *explicit* shame anxiety, where the subject is explicitly aware of their fear of humiliating exposure. In this explicit form, chronic shame is an explicit and conscious anticipation of acute shame; one feels a discrete rupture in social relations through a shame event that feels imminently possible.

However, as has also been discussed many experiences of chronic shame are *implicit* rather than explicit. As Bartky’s case study illustrates, this means that the subject *may not even be aware* or *may deny even to themselves* that they are anticipating shame. As noted above, DeYoung notes that some of her clients who suffer from chronic shame do not even know that they are anticipating and avoiding shame with debilitating frequency (DeYoung, 2015). Shame anticipation can become so deeply internalized that it has been theorized in psychotherapeutic literature as a shame “trait” or “disposition” (Leeming & Boyle, 2004: 376). While these terms are problematic because they put the onus on individuals to ‘heal’ rather than confronting the often structural basis for chronic shame, they give a sense of the deeply embedded nature of chronic shame in one’s experience of oneself and others. Instead of identifying shame as arising because of problematic social norms (such as the unjust stigma associated with HIV in Race’s example), individuals may come to feel that they themselves are inherently flawed. Rather than perceiving shame to be a central part of their lived experience, they instead live with chronic feelings of personal inadequacy. Writing about chronic shame as a result of lifelong and ongoing experiences of racism, Melissa Harris-Perry notes:

People who feel ashamed, or who are subjected to shaming experiences, tend to form chronically low opinions of themselves [and] tend to attribute bad outcomes to their own failures. They also tend to focus on negative information that reinforces the idea of their social unacceptability … In this way social rejection shapes experience of self and world. (Harris-Perry, 2011: 110)

When thinking about chronic shame as a political affect that disproportionally affects those living on the margins, who may have minority status or stigmatized identities, then it is important to recognize chronic shame not as dispositional, as though it is a property of an individual (Leeming & Boyle, 2004: 376), but instead to remain attentive to the way “that an ongoing difficulty with shame may be a product of [an] individual’s social and cultural niche” (Leeming & Boyle, 2004: 390), as a result of experiences like racism, stigma, social harms and social inequalities.

While shame is an intensely personal and individual experience, it only finds its full articulation within a rule and norm governed socio-cultural and political milieu. In this way, shame bridges our personal, individual, and embodied experience with the social and political world which contains us (Dolezal, 2015). It is because shame is *always* bound up in social and/or political norms that it can become politicized and oppressive. Indeed, chronic shame is often a result of power and domination that are experienced and propagated through affective and existential registers. Inhabiting a ‘world’ or identity that is rendered secondary and inferior by the dominant social order, and attempting to move into and find a space in the ‘world’ of the dominant other comes with an existential cost which is rendered salient in lived experience through an affective landscape. Understanding chronic shame and its phenomenology is key for elucidating its non-presence in experience. Being cognisant of chronic shame’s apparent invisibility is important because it has significant social and political consequences that can, and some argue, often do, remain unarticulated and unacknowledged.

The sort of subject that is constituted in light of the experience of chronic shame is one whose social and political horizons of possibility are compromised and constricted. Chronic shame can lead to a state of profound disempowerment where a toxic self-focus and self-interest drastically reduces the capacity for interpersonal empathy, responsibility and broader political and social engagement. As Retzinger notes, people experiencing covert shame may “function poorly as agents or perceivers” (Retzinger,1995: 1106). In fact, there is research which links the systematic shaming of certain individuals to political strategies of exclusion and marginalization, arguing that chronic forms of shame, which are induced by certain societal power relations, play a key role in the establishment and sustaining of social inequalities (Harris-Perry, 2011). As Pattison and his colleagues write:

Shame (…) is a curiously premoral or amoral state in which the self is inwardly engaged and preoccupied, paralysed either temporarily or permanently, and unable to engage in taking responsibility and judgement for its own actions; a failed, defiled, unwanted self cannot act as a responsive and responsible agent. Perhaps it is not surprising that a shamed person often feels speechless – they fall out of the community of human discourse and responsibility. (Sanders et al., 2011: 85)

In short, chronic shame, in both its explicit and implicit forms, has a largely negative and destructive potential, personally, socially and politically. Living with chronic shame shrinks one’s horizons of possibility with respect to interpersonal, social and political engagement, producing a sort of “social doubt,” where the social world presents obstacles rather than possibilities (Roberts, 2020). As a result, chronic shame seems to lack the positive potential for moral edification and social development that it is often discussed with relation to acute shame. Furthermore, living with chronic shame has material consequences in terms of one’s health and health outcomes, where evidence suggests that chronic shame experiences lead to a variety of negative health effects (Dolezal & Lyons, 2017; Harris-Perry, 2011).

## Conclusion

To conclude, the aim of this paper has been to think through a phenomenology of chronic shame, in both its explicit and implicit forms, demonstrating that this is an experience which is characterized largely by the absence of shame in experience. As we have seen, both explicit and implicit chronic shame are frequently characterised by the *absence* rather than the *presence* of shame. In these experiences, in differing manners, shame becomes a negative space around which experience is organized. Instead of the very recognizable searingly painful self-consciousness that accompanies episodes of acute shame, chronic shame can render shame invisible through implicit and explicit shame avoidance strategies. In this way, shame can be rendered invisible within social interactions and sometimes even to the self who is ‘experiencing’ it.

To address the challenges of identifying, diagnosing and describing the experience of chronic shame, and, concomitantly, attempting to describe its phenomenology, I have drawn from some conceptual tools within Edmund Husserl’s phenomenology, namely engaging with his formulation of the ‘horizon’. Husserl’s account of horizon has been deployed to think through how an absent presence, or non-presence, can nonetheless be a salient and important part of experience. While horizons are part of all experiences, the horizonal structure is particularly useful for theorizing aspects of the experience of chronic shame, an experience that is inherently characterized by present absences of shame. Understanding the horizonal structure of conscious experience hence, gives an important insight into the experiential structure of chronic shame. In particular, I have considered how chronic shame is characterised by structures of absence, anticipation and intersubjectivity, while playing a role in the formation of the character of one’s lifeworld. I have discussed the socio-political implications of chronic shame, where this experience is common in cases of systematic oppression, diminished social power and social domination. I have argued that living with chronic shame is inherently politically compromising and, because this experience is often invisiblized, understanding chronic shame and its phenomenology is of particular importance for accounts of intersubjectivity and social justice.

## Footnotes

1 Organizing one’s behaviour and interactions around the avoidance of shameful exposure is discussed at length in the stigma management literature, with its origins in Erving Goffman’s sociological analyses of stigma, interaction rituals and the presentation of self in everyday life. For example: (Goffman,1959, 1967, 1990).

2 I have drawn on this example from Kane Race in other writing. See: (Dolezal, 2021, 2022).

3 See Donald Nathanson’s ‘compass of shame’ for a more complete theorization of how shame is commonly bypassed for other compensatory experiences (Nathanson, 1992: 305–377). See also Sanderson’s discussion of shame avoidance strategies (Sanderson, 2015: 199–201).

4 As Susie Scott notes, experiences which are “unmarked” or characterised by “non-presence” are often sociologically neglected, which may be why experiences of chronic shame can remain undetected, invisible or difficult to articulate in socio-political analyses (Scott, 2018).

5 Italics in original.

6 For instance, Marta Jorba offers an analysis of cognitive horizons, applying Husserl’s account of horizons to illustrate cognitive affordances (Jorba, 2020).

7 Theorizing the experiences of living between ‘worlds’ preoccupies the US Latina feminist philosophical tradition, with thinkers such as Linda Martín Alcoff (Alcoff, 2006), Ofelia Schutte (Shutte, 1998), Marian Ortega (Ortega, 2016) and Maria Lugones (Lugones, 2003) all writing about the textures of lived experiences of having a marginal identity (Latina) and the existential cost of fitting in to the dominant social order (the Anglo/US white world). Maria Lugones theorizes the experience of ‘world’-traveling in her seminal 1987 *Hypatia* article “Playfulness, ‘World’-traveling and Loving Perception” writing: “A ‘world’ in my sense my be an actual society, including its dominant culture’s description and construction of life, including a construction of the relationships of production, of gender, race, etc. But a ‘world’ can also be such a society given a nondominant, a resistant construction, or it can be such a society given an idiosyn-cratic construction … it is problematic to say that these are all constructions of the same society. But they are different “worlds” … A ‘world’ need not be a construction of a whole society. It may be a construction of a tiny portion of a particular society. It may be inhabited by just a few people. Some ‘worlds’ are bigger than others” (Lugones, 2003: 87f.). Hence, the term ‘world,’ as Lugones deploys it here can be understood as a shared frame of reference, “community of meaning” (Ortega, 2016: 65) or shared “world horizon,” to use phenomenological terminology, where the norms, tacit assumptions, customs, embodied practices belong to a dominant group who ‘belong’ to that world, and feel ‘at home’ there. There are multiple worlds that overlap and intersect, and we can inhabit many simultaneously.
